# Honeybees Tolerate Cyanogenic Glucosides from Clover Nectar and Flowers

**DOI:** 10.3390/insects9010031

**Published:** 2018-03-13

**Authors:** Antoine Lecocq, Amelia A. Green, Érika Cristina Pinheiro De Castro, Carl Erik Olsen, Annette B. Jensen, Mika Zagrobelny

**Affiliations:** Department of Plant and Environmental Sciences, University of Copenhagen, Thorvaldsensvej 40, DK-1871 Frederiksberg C, Copenhagen, Denmark; amelia.a.green@gmail.com (A.A.G.); erca@plen.ku.dk (É.C.P.D.C.); ceo@plen.ku.dk (C.E.O.); abj@plen.ku.dk (A.B.J.); miz@plen.ku.dk (M.Z.)

**Keywords:** clover (*Trifolium repens*), cyanogenic glucoside, honeybee (*Apis mellifera*), linamarin, nectar

## Abstract

Honeybees (*Apis mellifera*) pollinate flowers and collect nectar from many important crops. White clover (*Trifolium repens*) is widely grown as a temperate forage crop, and requires honeybee pollination for seed set. In this study, using a quantitative LC-MS (Liquid Chromatography-Mass Spectrometry) assay, we show that the cyanogenic glucosides linamarin and lotaustralin are present in the leaves, sepals, petals, anthers, and nectar of *T. repens*. Cyanogenic glucosides are generally thought to be defense compounds, releasing toxic hydrogen cyanide upon degradation. However, increasing evidence indicates that plant secondary metabolites found in nectar may protect pollinators from disease or predators. In a laboratory survival study with chronic feeding of secondary metabolites, we show that honeybees can ingest the cyanogenic glucosides linamarin and amygdalin at naturally occurring concentrations with no ill effects, even though they have enzyme activity towards degradation of cyanogenic glucosides. This suggests that honeybees can ingest and tolerate cyanogenic glucosides from flower nectar. Honeybees retain only a portion of ingested cyanogenic glucosides. Whether they detoxify the rest using rhodanese or deposit them in the hive should be the focus of further research.

## 1. Introduction

Honeybees (*Apis mellifera*) pollinate many important crop species. More than 76% of the top 115 global food crops rely on animal pollination [1]. The value of pollination services provided by honeybees and wild pollinators has been estimated at over $215 billion worldwide [2]. Bees and flowering plants have a mutualistic relationship: the bees pollinate the plants and the plants provide nutrition to the bees in the form of nectar and pollen. Floral nectar, stored as honey, is the main energy source for individual honeybees and for the colony. Protein-rich pollen provides most of the nutrients required for honeybees’ physiological development, including essential amino acids, lipids and vitamins [3]. Both nectar and pollen can also contain a range of plant secondary compounds [4]. Cyanogenic glucosides (CNglcs) are secondary metabolites widespread in plants. CNglcs act as a plant defense: when a stabilizing glucose molecule is removed from the compounds by a catalyzing β-glucosidase enzyme, toxic hydrogen cyanide (HCN) is released and inhibits cellular respiration. Herbivores can overcome plant defensive compounds by detoxification, excretion or sequestration [5], and can even use these compounds in their own defense against natural enemies [6]. The same compounds may also be found in nectar and pollen, where they are unlikely to deter beneficial insects such as bee pollinators [7]. Molecular analyses have revealed that honey samples from several European locations contain DNA from CNglc-containing plants such as clover [8,9], indicating that honeybees collect nectar and/or pollen from these plants. Low concentrations of plant defensive compounds in nectar seem to have beneficial effects for bees. For example, honeybees preferentially feed on nectar with a low caffeine concentration, but are deterred by a high concentration [10], a phenomenon known as hormesis [11]. High nectar concentrations of the cyanogenic glucoside amygdalin cause “malaise” symptoms in honeybees [12,13], but the concentrations of amygdalin naturally found in nectar do not deter honeybee feeding or otherwise affect their behavior [14,15,16]. Since β-glucosidases are present as digestive enzymes in honeybees [17], it is possible that any ingested CNglcs could be degraded to release HCN. Two methods of detoxifying HCN have been shown in living organisms: rhodanese activity is found mainly in vertebrates, while β-cyanoalanine synthase is found in plants and many insects [18].

White clover (*Trifolium repens*) is a leguminous, cyanogenic crop grown worldwide to fix nitrogen in soil and as forage for mammals and honeybees [19]. It is largely self-incompatible, requiring pollination for seed production [20]. A study in Australia [21] found that honeybees comprised 88% of visitors to white clover plants, with seed yield increased 30 fold by bee activity. Several clover species produce large quantities of nectar suitable for honey production [22]. The leaves of *T. repens* and other clover species [23,24] contain various quantities and ratios of the CNglcs linamarin and lotaustralin. Despite the importance of pollination, the CNglc levels in clover nectar and flowers have not previously been measured. However, the presence of amygdalin in nectar and pollen of almond trees [25], and linamarin and lotaustralin in the nectar of *Lotus japonicus* [26], suggests that CNglcs are likely to be found in clover nectar.

In this study, we describe and measure the concentrations of naturally occurring CNglcs found in white clover leaves, flower parts and nectar. We assess the effect of CNglcs on the survival of honeybees by feeding them sugar solutions spiked with different concentrations of CNglcs. Finally, we determine whether bees have the ability to degrade and/or detoxify CNglcs in enzyme activity assays.

## 2. Materials and Methods

### 2.1. Plant Material

Clover seeds were obtained from the USDA-ARS-GRIN plant germplasm collection (http://www.ars-grin.gov/): *Trifolium repens* PI 205062, PI 668016. Plants were grown in standard greenhouse conditions at the University of Copenhagen, Frederiksberg. Leaf samples were collected in microfuge tubes, frozen immediately in liquid nitrogen, weighed after freezing, and kept at −80 °C until further analyses. Flowers were separated into sepals, petals and reproductive organs (stamens and pistils combined). Anthers were also sampled separately from other flowers in the same inflorescence. Parts from approximately 20 flowers were combined per microfuge tube. Flower part samples were frozen in liquid nitrogen, weighed and stored at −80 °C until further analyses.

### 2.2. Nectar Sampling

Nectar was collected from each clover flower by inserting a gel-loading pipette tip with an elongated, narrow end (Corning: size 0.2–10 μL; length 83 mm; diameter at end of tip c. 0.4 mm) into the corolla opening without damaging the flower tissues, and pipetting the nectar into a microfuge tube (method adapted from [26]). For each sample, nectar was pooled from all flowers of one inflorescence, giving 1–5 μL of nectar per sample. Samples were frozen in liquid nitrogen and kept at −80 °C until further analyses.

### 2.3. Bee Material 

Bees were obtained from hives kept at the University of Copenhagen, Frederiksberg. Brood frames were collected from three separate colonies and kept in an incubator at 34 °C. Newly emerged worker bees from the brood frames were mixed together upon emergence and transferred to experimental cages made from standard transparent plastic cylindrical honey pots (450 mL) with a height of 8.5 cm and a diameter of 9 cm.

### 2.4. Chronic Feeding of Cyanogenic Glucosides 

Seven cup-shaped hoarding cages (450 mL) each containing twenty recently emerged bees were prepared. All cages were provided with a filter paper in the bottom. Ad-libitum 50% sucrose solutions including 1, 10, or 100 ng/μL amygdalin or linamarin, plus a control cage with only 50% sucrose solution, were provided in graduated (1 mL) syringes. All cages were kept in an incubator at 34 °C. Mortality and sucrose consumption were recorded daily. The experiment ran for 22 days. All bees surviving at the end of the experiment were frozen at −20 °C and later analyzed for the presence of CNglcs.

### 2.5. LC-MS Analysis 

Before preparation for LC-MS analysis, samples were quantified by weight [27] for bees, leaves and flower parts; or by volume (μL) for nectar. Plant samples (with the exception of nectar) were then boiled in either 55% (*v*/*v*) or 85% (*v*/*v*) Methanol solution containing 0.1% formic acid, and 0.044 mM amygdalin (as internal standard), for 5 min in a water bath, cooled on ice, and homogenized as described in [26]. Nectar samples were prepared by mixing with water to a total volume of 30 μL. Bees were ground up in ice-cold 55% (*v*/*v*) Methanol containing 0.1% formic acid and 0.044 mM amygdalin (as internal standard); except for bees that had ingested amygdalin, which were processed without the internal standard. All bee and plant samples (except nectar) were subsequently passed through an Anopore 0.45 μm filter (Whatman, Maidstone, UK). Analytical LC-MS was carried out using an Agilent 1100 Series LC (Agilent Technologies, Waldbronn, Germany) interfaced with a Bruker HCT-Ultra ion trap mass spectrometer (Bruker Daltonics, Bremen, Germany). Chromatographic separation was carried out using a Zorbax SB-C18 column (Agilent; 1.8 μM, 2.1 × 50 mm) at a flow rate of 0.2 mL/min. The oven temperature was maintained at 35 °C. The mobile phases were as follows: A, H_2_O with 0.1% (*v*/*v*) HCOOH and 50 μM NaCl; B, MeCN with 0.1% (*v*/*v*) HCOOH. The gradient program was as follows: 0 to 0.5 min, isocratic 2% B; 0.5 to 7.5 min, linear gradient 2 to 40% B; 7.5 to 8.5 min, linear gradient 40 to 90% B; 8.5 to 11.5 isocratic 90% B; 11.6 to 17 min, isocratic 2% B. The flow rate was increased to 0.3 mL/min in the interval 11.2 to 13.5 min. The mass spectrometer was run in positive electrospray mode. Mass spectral data were analyzed with the native data analysis software. Sodium adducts of linamarin (retention time (RT) 2.6 min, [M + Na]^+^ at *m*/*z* 270), lotaustralin (RT 5.5 min, [M + Na]^+^ at *m*/*z* 284), and amygdalin (RT 6.6 min, [M + Na]^+^ at *m*/*z* 480) were detected and their RTs compared to authentic standards produced in our laboratory [28]. The total amount of each compound was estimated based on its Extracted Ion Chromatogram (EIC) peak areas and quantified based on calibration curves of linamarin, lotaustralin, and amygdalin standards.

### 2.6. β-Glucosidase Activity Assay 

HCN emission from crops, guts and whole worker bees collected directly from the hives in spring was analyzed by crushing 10 samples from each sample type individually in a microfuge tube with 200 μL (crops and guts) or 500 μL (whole bees) 60 mM citric acid buffer (pH 6) containing either 1 mM linamarin or 1 mM amygdalin as substrate. A PCR-tube containing 200 μL 1 M NaOH was inserted into the microfuge tube containing the reactants to trap released CN^−^. The mixture was incubated for 90 min (whole bees) or 180 min (crop and guts) at 30 °C. Incubation times were based on the time needed for exhaustive CNglc degradation if an effective β-glucosidase was present in the sample [29]. HCN emitted from samples during incubation was trapped in the NaOH solution in the PCR tube, and analyzed colorimetrically as previously reported [30]. Quantification was based on a corresponding standard curve with known amounts of KCN dissolved in 60 mM citric acid buffer. Positive controls were linamarin + linamarase (β-glucosidase from Cassava, extracted in our lab) and amygdalin + emulsin (β-glucosidase from almond, Sigma No. G-8625, EC 3.2.1.21, Copenhagen, Denmark), and negative controls were bees or bee tissues without added substrate as well as substrates without added bees. Bee crop and guts were not emptied of contents before analysis since they had not fed on flowers containing CNglcs prior to this experiment.

### 2.7. Genes Involved in Degradation and Detoxification of Cyanogenic Glucosides in the Honeybee Genome 

To analyze whether bees have enzymes able to degrade and/or detoxify CNglcs, tBLASTn searches in the bee genomes in GenBank (as of February 2018) were carried out with default settings. The following β-glucosidase, rhodanese and β-cyanoalanine synthase enzyme sequences from different insect species were used as query sequences: *Bombyx mori* (XP 012545628.1, AK385118.1, XM_004931107.2), *Plutella xylostella* (XM_011559276.1, XM_011556416.1, XM_011557735.1), *Zygaena filipendulae* (LT635663.1, MF038027.1 and unpublished sequences from transcriptomes—see [31,32], and spider mite *Tetranychus urticae* (KF981737.1, XP_015783585.1).

### 2.8. Statistical Analysis 

Student’s t-tests (conducted using Excel) were used to determine whether the averages of HCN emission from bee guts and whole bees were significantly larger than the averages of control samples. Graphs were constructed using MS Excel (Microsoft, Redmond, WA, U.S.A.). The effect of chronic feeding of CNglcs on honeybee survival was assessed by carrying out a Kaplan–Meier survival analysis with treatment as a factor variable using IBM SPSS Statistics version 23 (IBM Corporation, Armonk, NY, USA).

## 3. Results

### 3.1. Cyanogenic Glucosides in White Clover 

**Figure 1:**
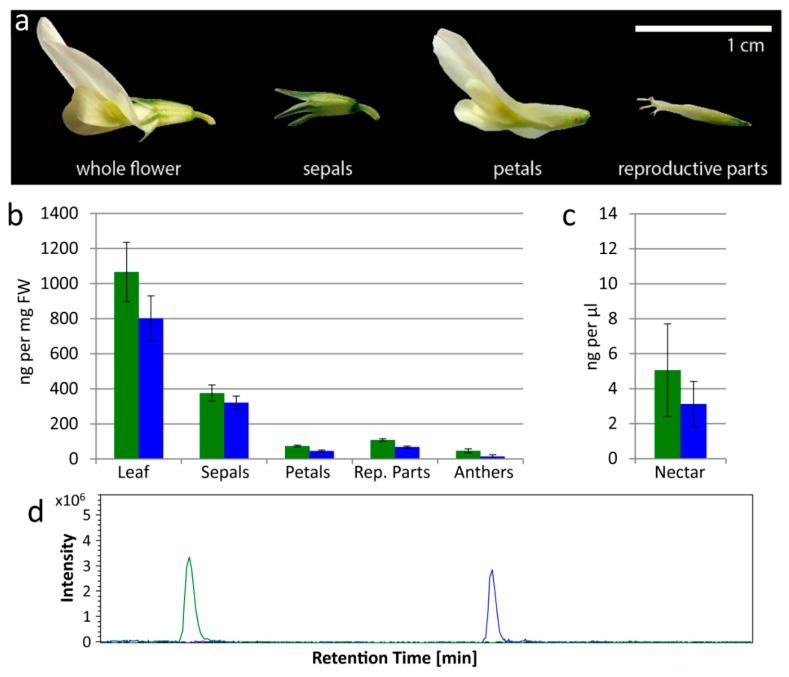
Cyanogenic glucosides are found by LC-MS in leaves, flowers and nectar of white clover. (**a**) A single *Trifolium repens* flower, separated into sepals, petals and reproductive parts (stamens and pistils combined). (**b**) Linamarin (green) and lotaustralin (blue) concentrations (determined by LC-MS) in leaves and flower parts (sepals, petals, combined reproductive parts, anthers) of *T. repens*; four samples for each. (**c**) Linamarin (green) and lotaustralin (blue) concentrations (determined by LC-MS) in nectar of *T. repens*; four samples. Error bars in (**b**,**c**) show standard error. (**d**) Representative Extracted Ion Chromatogram (EIC) from LC-MS showing linamarin (green, [M + Na]^+^ = 270) and lotaustralin (blue, [M + Na]^+^ = 284) in nectar.

Flower parts were examined separately: sepals, petals, reproductive parts (stamens and pistils combined) and anthers (which comprise mainly pollen) (Figure 1a,b). Leaf samples were also examined to confirm that all plants contained CNglcs (ng per mg FW). Mean (±s.e.) linamarin content in leaves was 1066.2 ± 169.4 ng/mg; and in flower parts 375.2 ± 45.9, 73.7 ± 5.2, 107.6 ± 7.8, and 46.0 ± 11.3 ng/mg in sepals, petals, reproductive parts and anthers respectively (Figure 1b). Mean lotaustralin content in leaves was 800.3 ± 129.6 ng/mg; and in flower parts 320.9 ± 37.7, 45.3 ± 5.4, 68.2 ± 5.4, and 14.2 ± 9.6 ng/mg in sepals, petals, reproductive parts and anthers respectively (Figure 1b). Nectar was also analyzed by LC-MS to determine the CNglc content (ng per μL) (Figure 1c,d). In nectar, mean (±s.e.) linamarin content was 5.1 ± 2.7 ng/μL, and lotaustralin content was 3.1 ± 1.3 ng/μL (Figure 1c). Since we found both linamarin and lotaustralin in all the flower components analyzed, including nectar, these compounds are not exclusively found in vegetative tissue in clover (Figure 1).

### 3.2. Honeybee Survival and Consumption 

**Figure 2:**
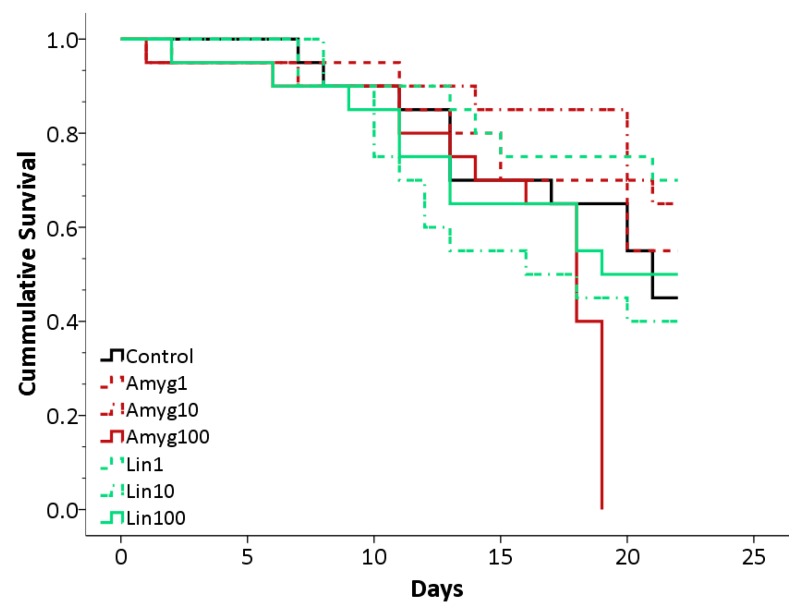
Survival plot for bees in the control, amygdalin and linamarin treatments. Amygdalin treatments in red, Linamarin in green and Control represented by a black line. Concentrations of 1 ng/μL shown by a dashed line, 10 ng/μL shown by dots and dashes and 100 ng/μL a full line.

Five out of the six cages, fed different concentrations of linamarin or amygdalin, showed survival rates largely indistinguishable from the control cage after 22 days, but one cage (fed 100 ng/μL amygdalin; mean (±S.E.) lifespan 15.7 ± 1.1 days) showed a sudden, significant decline on day 19 (Log Rank Test: χ^2^ = 26.03; d.f = 6; *p* < 0.001; Figure 2). Mean survival for all other treatments were as follows: 18.1 ± 1.1 days (mean ± S.E.) for control bees, 18.4 ± 1.2 days and 19.4 ± 1.2 days for amygdalin 1 and 10 respectively; 19.2 ± 1.1 days, 16.2 ± 1.2 days and 17.0 ± 1.4 days for linamarin 1, 10 and 100 respectively. The decline observed in the amygdalin 100 treatment could have been due to several factors, given that it affected only one cage. Replications of the experiment would be required to test if the decline was linked solely to the unusually high concentration of amygdalin. On average, a bee in the control cage drank 28.3 ± 2.1 μL sucrose per day (mean ± S.E.). In the linamarin 1, 10 and 100 treatments, the average was 23.8 ± 2.0 μL, 21.4 ± 1.7 μL and 17.8 ± 2.0 μL per day respectively. In the amygdalin 1, 10 and 100 treatments, a bee drank an average of 25.1 ± 2.1 μL, 23.8 ± 1.7 μL and 23.1 ± 3.0 μL per day. The group fed 100 ng/μL linamarin appeared to consume less sugar solution than bees from the control treatment, although further replications would be required to determine the extent of this effect. 

The average FW of a surviving bee, after feeding with CNglcs for 22 days, was 113 ± 12 mg. We found CNglcs in over 80% of samples (*n* = 15) analyzed; 0.21 ± 0.12 ng linamarin per mg FW and 0.20 ± 0.49 ng amygdalin per mg FW for ingestion of sugar solution with 10 ng/μL, and 4.37 ± 4.28 ng linamarin/mg FW for ingestion of sugar solution with 100 ng/μL (mean ± S.E.). We were unable to carry out this analysis for the amygdalin 100 treatment due to the last bee dying on day 19. Based on our daily consumption results, it appears that each bee consumed c. 210–250 ng CNglc per day (c. 4.7–5.3 μg CNglc in total over 22 days) when fed on 10 ng/μL linamarin or amygdalin solution, and c. 1700–2400 ng CNglc per day (c. 39–51 μg CNglc in total over 22 days) when fed on 100 ng/μL linamarin solution. 

### 3.3. Degradation and Detoxification of Cyanogenic Glucosides by Bees 

One to several β-glucosidase genes were found in honeybee species (XP 016768291.1 in *Apis mellifera*) with 40–45% sequence identity on the amino acid level compared to query sequences. Two methods of detoxifying HCN have been shown in living organisms: rhodanese activity is found mainly in vertebrates, while β-cyanoalanine synthase is found in plants and many insects. One rhodanese gene was found in each bee species (XP 001120855.1 in *Apis mellifera*) with 40–47% sequence identity compared to query sequences. However, β-cyanoalanine synthase was not present in the bee genomes, the most closely related genes were cystathionine beta-synthases, but with a sequence identity of only 33–35% (NP 001035353.1 in *Apis mellifera*). 

To analyze if the bee β-glucosidase enzymes were capable of degrading CNglcs, HCN emission from homogenized whole honeybees with added linamarin or amygdalin was measured (Table 1), showing that homogenized bees degrade both linamarin and amygdalin. The HCN emission from bees homogenized with amygdalin was significantly higher than from bees without added substrate (*p* = 0.0434, Student’s *t-*test). The HCN emission from bees homogenized with linamarin was not significantly higher than from bees without added substrate (*p* = 0.1296, Student’s *t-*test), although all samples had higher emission than the control, indicating a clear trend. Positive controls of substrates with specific β-glucosidases gave a much higher HCN emission (emulsin: 60 nmol and linamarase: 11 nmol, from 500 nmol substrate added). We also tested HCN emission from dissected crops and guts of bees to see if the enzyme activity found in whole bees originated in these tissues. Crops had no activity, but guts did (Table 1), and HCN emission from guts homogenized with linamarin or amygdalin were both significantly higher than from guts without added substrates (*p* = 0.0118 and *p* = 0.0056 respectively, Student’s *t-*test). 

## 4. Discussion

In this study, we report the presence of linamarin and lotaustralin throughout leaves, flowers and nectar of white clover, a forage crop pollinated by honeybees [21,33]. Since honeybees are important pollinators of clovers and many other cyanogenic plants, we examined the effects on honeybees of ingesting CNglcs found in nectar. We found that honeybees do not appear to suffer lethal adverse effects from feeding on artificial nectar (sucrose solution) containing naturally occurring concentrations of linamarin or amygdalin (1–10 ng/μL), although our results indicated that the presence of CNglcs may alter the bees’ daily sugar consumption. Throughout the experiment, treatment bees consumed on average between 12 and 38 percent less sugar solution than control bees. This may indicate altered metabolic stress, and bees may reduce their consumption of CNglc-containing solutions to avoid post-ingestive toxicity [12]. Further experiments are required, with more replicates of different CNglc concentrations, to determine whether high concentrations of CNglcs affect honeybee survival or feeding patterns.

From this study, it is evident that honeybees can take up linamarin and amygdalin intact during feeding and retain the compounds in their bodies. Accordingly, ingested CNglcs do not appear affected by the β-glucosidase activity found in whole bees and bee guts. The β-glucosidase enzymes are probably either compartmentalized away from ingested CNglcs in the gut or ineffective during digestion. Our results allowed us to estimate and compare the average amount of CNglcs ingested per bee over 22 days with the amount of CNglcs retained in the whole bee after 22 days of feeding measured by LC-MS. We found that honeybees retained on average only a small proportion of the amount of CNglcs ingested (<26% of the CNglcs consumed on average per bee per day), although a few individual bees contained more than double the average corresponding to more than half a day’s consumption. This suggests that bees may degrade and/or detoxify CNglcs. The enzyme hypothesized to be the primary HCN detoxification enzyme in insects, β-cyanoalanine synthase, was not present in the bee genomes in GenBank (as of February 2017). We cannot rule out that bees could have recruited a different enzyme for CNglc detoxification, or that microbes in the bee gut or in stored nectar and bee bread [34,35] could degrade and/or detoxify CNglcs. We suggest that, in a natural setting, bees keep CNglcs intact in the crop and deposit them in the hive, and/or detoxify them using rhodanese or enzymes from microbes. 

CNglcs have previously been shown to have antimicrobial properties towards plant pathogens [36] but little is known about whether CNglcs are retained by bees for defense against pathogens. It is possible that honeybees use secondary plant compounds from nectar and pollen for prophylactic disease control or as self-medication: bees consuming nectar containing plant secondary metabolites have previously been shown to have a reduced pathogen load or parasite infections [37,38,39]. Several types of honey have shown antimicrobial effects against honeybee diseases [40], and adult honeybees select honey types (likely containing different plant secondary metabolites) depending on their health status [41]. Future studies will show if honeybees feed CNglcs to their offspring or build them into their hives to protect them from disease.

## 5. Conclusions

Our study shows that the cyanogenic glucosides linamarin and lotaustralin are present in the leaves, sepals, petals, anthers and nectar of white clover plants. We also find that honeybees can survive ingestion of cyanogenic glucosides found in nectar, and retain cyanogenic glucosides in their bodies after ingestion. Only a small proportion of ingested cyanogenic glucosides are retained, suggesting that honeybees can degrade or detoxify cyanogenic glucosides, although the absence of β-cyanoalanine synthase in the honeybee genome indicates that the detoxification process is different from most insects. Future investigations should elucidate the mechanism for detoxification and establish how honeybees use cyanogenic glucosides for defense against pathogens.

## Figures and Tables

**Table 1:** β-glucosidase activity in whole bees and bee gut determined by measuring hydrogen cyanide (HCN) emission from homogenized whole bees and bee gut with nothing (control), linamarin or amygdalin added (average total nmol HCN emitted during incubation for each sample (± standard deviation)). Whole bees (*n* = 10) and bee guts (*n* = 10) were tested in two different experiments.

HCN Emission	β-Glucosidase Activity in Whole Bees			β-Glucosidase Activity in Bee Gut		
	Linamarin	Amygdalin	Control	Linamarin	Amygdalin	Control
HCN emission (nmol ± s.d.)	2.9 (±1.6) ^a^	7.1 (±3.0) ^a^	0.5 (±0.2) ^a^	6.6 (±1.9) ^a^	5.3 (±1.2) ^a^	0.3 (±0.0) ^a^

^a^ *n* = 10.
